# Addressing the needs of people with extensively drug-resistant TB through pre-approval access to drugs and research

**DOI:** 10.5588/pha.23.0033

**Published:** 2023-12-07

**Authors:** J. Stillo, M. Frick, J. Galarza, S. Kondratyuk, A. Makone, L. McKenna, W. Vandevelde, P. Winarni, P. Agbassi

**Affiliations:** 1 Department of Anthropology, Wayne State University, Detroit, MI, USA; 2 Global Tuberculosis Community Advisory Board, New York, NY, USA; 3 Treatment Action Group, New York, NY, USA; 4 McGovern Medical School at the University of Texas Health Science Center, Houston, TX, USA; 5 Department of Political Science, Stellenbosch University, Cape Town, South Africa; 6 GNP+, Cape Town, South Africa

**Keywords:** compassionate use, XDR-TB, novel TB drugs

## Abstract

Multiple therapeutic options exist for people with drug-resistant TB (DR-TB), but there is an urgent need to improve access to novel compounds and regimens for people with difficult to treat forms of TB. In additional to formal research studies and clinical trials, other mechanisms of accessing promising new TB compounds need to be introduced as soon as these drugs have shown efficacy and safety in phase II trials. Pre-approval access programs for newer TB drugs such as bedaquiline, delamanid, and pretomanid all suffered from shortcomings. These can be addressed for the next generation of new TB drugs through a series of concerted actions by stakeholders at multiple levels. In this viewpoint, we advocate for transparent, accessible pre-approval access as a core element of person-centered care for DR-TB.

## Abstract (fr)

Il existe de nombreuses options thérapeutiques pour les personnes atteintes de TB résistante aux médicaments (DR-TB), mais il est urgent d’améliorer l’accès aux nouvelles molécules et aux nouveaux schémas thérapeutiques pour les personnes atteintes de formes de TB difficiles à traiter. Outre les études de recherche formelles et les essais cliniques, d’autres mécanismes d’accès aux nouvelles molécules prometteuses contre la TB doivent être mis en place dès que ces médicaments ont démontré leur efficacité et leur innocuité lors des essais de phase II. Les programmes d’accès avant approbation pour les nouveaux médicaments contre la TB tels que la bédaquiline, le delamanid et le pretomanid ont tous souffert de lacunes. Ces problèmes peuvent être résolus pour la prochaine génération de nouveaux médicaments contre la TB grâce à une série d’actions concertées entre les parties prenantes à différents niveaux. Dans cette optique, nous préconisons un accès transparent et accessible avant approbation, en tant qu’élément central des soins centrés sur la personne pour la DR-TB.

Of the 10 million people who become ill with TB each year, almost half a million have drug-resistant forms of disease (DR-TB).^1^ These individuals have limited treatment options, especially if they are sick from strains of TB that have additional resistance to second-line medications such as the fluoroquinolones, bedaquiline (BDQ) and/or linezolid. After almost a half-century of stagnation, notable advances in TB drug development are being made.^2^ Three new agents—BDQ, delamanid (DLM), and pretomanid (Pa)—are now globally recommended, approved by stringent regulatory agencies, and being used as part of novel regimens for the treatment of DR-TB.^3^ An examination of the TB drug pipeline shows multiple new compounds in various stages of development.^4^ Although the pace of trials has not kept up with the pressing global need for new drugs and treatment regimens, there is reason for optimism.

While cure rates for DR-TB have vastly improved as new and re-purposed agents and shorter regimens have been rolled out, there are still people who are not cured even with optimal existing treatment regimens. Data from high-burden countries such as India and South Africa, for example, show that there are rising rates of *Mycobacterium tuberculosis* resistance to drugs such as BDQ, LZD, and the fluoroquinolones,^5^,^6^ cornerstones of current optimal treatment regimens recommended by the WHO for DR-TB. People with these extensively drug-resistant TB (XDR-TB; defined as multidrug-resistant *M. tuberculosis* with additional in vitro resistance to a fluoroquinolone and one of the other two group A drugs,^7^ according to the 2021 WHO definitions) strains have limited treatment options. These individuals are a small but growing population and are often treated as inpatients for 18–24 months with rescue regimens containing drugs with limited efficacy and poor tolerability, such as amikacin, ethionamide, para-aminosalicylic acid, and the carbapenems.^8^ Additionally, timely access to drug susceptibility testing to guide therapy is still not widely available. As a result of these circumstances, treatment success rates in this population are likely to be well below 50%, harkening back to the days before BDQ was available, when people with XDR-TB faced the very real possibility of having ‘untreatable’ TB and all its attendant consequences.

## ACCESS TO NOVEL TUBERCULOSIS COMPOUNDS THROUGH TRIALS

To meet the needs of people with XDR-TB, we need formal clinical trials designed to optimize treatment duration and composition. Several TB drugs with new mechanisms of action are already or soon to be in phase IIb trials, including those targeting mycobacterial cell wall synthesis (quabodepistat [formerly OPC-167832], TBA-7371, BTZ-043), energy synthesis and respiration (telacebec [formerly Q203]), and protein synthesis (GSK-656). However, the research networks and other collaborative initiatives that will take these compounds forward are all planning to study these new drugs (at least initially) on top of a BDQ and a DLM or Pa backbone.^4^ People with XDR-TB require novel drugs to be studied in novel combinations that do not include drugs their strains of TB have resistance to. Animal models and pre-clinical and translational evidence should guide the design of these formal trials.

## PRE-APPROVAL ACCESS TO NOVEL TUBERCULOSIS COMPOUNDS OUTSIDE OF TRIALS

For people with XDR-TB who might be unable to participate in such clinical trials, we need to provide more equitable care through rapid and systematic access to novel TB drugs via pre-approval access programs—or what are sometimes referred to as ‘compassionate use’ programs.^9^ Pre-approval access programs are a mechanism for people with XDR-TB to gain access to experimental drugs/regimens with potential therapeutic benefits prior to those drugs being approved by the stringent or other regulatory agencies with authority to approve them in different settings. Pre-approval access programs have been successful in improving the care of individuals with limited treatment options for other serious health conditions.^10^ Such programs can take several forms and are different from ‘pilot’ or ‘roll out’ programs, as the drugs being accessed have not yet been registered for the indication for which they are being used. Examples include 1) individual access on a ‘named-patient basis’ through participating clinicians; 2) group access through participating centers via bulk importation; or 3) single-arm clinical trials in which people with limited therapeutic options are offered the medications as part of clinical cohort studies. Although different in their structure, composition, and funding, each of these meets the definition of a pre-approval access program.

Pre-approval access programs should be seen as a shared responsibility between governments, global health actors (including policy and funding agencies), and drug developers. While such programs are designed to consider the needs of, and primarily to benefit, individuals with limited therapeutic options and the TB programs and clinicians responsible for their care, these programs can also benefit companies and drug sponsors, and support policy making. They do so by providing an opportunity to familiarize providers with new treatments before they are approved, as was described for the BDQ pre-approval access program in South Africa.^11^ Close monitoring and reporting of additional safety data on novel compounds and regimens—especially when such data are collected at a supranational level—can help to guide their broader use among populations of patients who might be excluded from formal clinical trials.

## EXPERIENCE WITH PRE-APPROVAL ACCESS IN TUBERCULOSIS

Unfortunately, when it comes to TB, pre-approval access has been limited at best.^11^ BDQ, the first novel TB drug from a new class in 40 years, became available shortly after the phase IIb trial was completed. The pre-approval access program by Johnson & Johnson (New Brunswick, NJ, USA) was started early but slowly due, in part, to concerns about BDQ’s safety that emerged from the phase IIb study. In some settings, BDQ did become available prior to regulatory approval through bulk ‘donation’ and operational research programs in which cohorts of people with DR-TB were enrolled and closely monitored.^12^ Such programs improved access to BDQ prior to its approval, but only a small number of people living in specific geographic settings were able to benefit from the program. Pre-approval access to DLM was limited by the fact that its parent company (Otsuka; Tokyo, Japan) opened the program 3 years into the phase III trial and the strict and conservative access criteria. When Otsuka was lobbied to provide broader access to the DLM, the company cited safety concerns about combining it with BDQ, as they both had the potential to prolong the QTcF interval.^13^ Although the company did develop a mechanism for pre-approval access on a named-patient basis, the program was not publicized and there was limited uptake. The program also did not begin until after DLM was conditionally registered by the European Medicines Agency, which meant no European Union countries could access the drug in this way, even those with a relatively high burden of TB (e.g., Romania). Pre-approval access to Pa was not initially available through the drug’s sponsor, the TB Alliance, due, in part, to a lack of funding.^14^ The TB Alliance’s first commercial partner for Pa (Viatris; Canonsburg, PA, USA) did eventually launch a pre-approval access program but with limited success, given the relatively late timing and operational errors like charging programs for pre-approval access to the medication in some settings.^15^

The relative inexperience of the companies and sponsors who controlled access to BDQ, DLM, and Pa was exacerbated by the limited experience and knowledge of national TB programs in offering drugs through pre-approval access mechanisms, deepening shortcomings and inequities in pre-approval access to these three medicines. Limiting access further was a paradigm of protectionism in TB, where providers and policymakers (the people who made key decisions about the care of people with TB) advocated for rationing drugs out of fear of developing resistance and ‘wasting’ the new medications. There was also concern expressed that negative experiences with a drug given as part of pre-approval access could later harm the subsequent chances of formal registration.^16^ These multiple factors led to a dire access landscape for people with DR-TB who had limited treatment options and often led to irrational national policies about newer drug use, reflecting, in part, the drug rationing approach sanctioned by WHO guidelines.^17^ Without intervention some of these mistakes will inevitably be repeated with the next generation of novel drugs that are making their way through the TB pipeline.

The new drugs with novel mechanisms of action already or soon to be in phase IIb trials are sponsored by companies and organizations with varying degrees of experience and track records of successful pre-approval access programs. For example, quabodepistat is sponsored by Otsuka, whereas the other DprE1 inhibitors and some of the other novel compounds are sponsored by public universities (Ludwig Maximilian University, Munich, Germany) or non-profit product development partnerships (the TB Alliance). These organizations will need support to develop robust pre-approval programs. Such programs must address important aspects in terms of timing, eligibility criteria, structure, availability, financing, consent, and transparency (see Table 1). Positive models could be adapted from other disease paradigms, including pre-approval access programs for antiretroviral therapy. For example, the ‘parallel track protocol’ mechanism established for stavudine in the 1990s not only allowed thousands of people to access this medication before it was approved, but also established a safer daily dose for the drug to reduce the risk of neuropathy.^18^ The National Clinical Access Programs for BDQ and DLM in South Africa and the named-patient access program for pediatric formulations of DLM also offer positive examples that could be refined and replicated for newer TB drugs.^19^

**TABLE 1:** Key considerations in pre-approval access programs for DR-TB

Component	Consideration
Timing of program	As soon as phase IIb data are available showing a favorable risk-benefit profile.
Eligibility criteria	Should be based on unmet medical need and allow for the inclusion of people who might not be included in trials. Should consider specific safety data (if they are available), potential for drug-drug interactions, and availability of companion agents, to name a few considerations. These will likely differ depending on the characteristics of the compound but should be clearly specified
Backbone regimen	Should require a minimum number of effective medications and allow for combination with other new/experimental drugs available via pre-approval process programs
Structure of program	Should harmonize with national legal and regulatory environments
Availability	Should emphasize global, regional, national, and sub-national (local) equity
Financing	Depending on the model, should be the responsibility of the drug company/sponsor, donors, and/or national governments and not be a financial burden on clinicians or on people with TB
Consent	Should be robust and meaningful, protecting vulnerable populations while offering improved access. Should specify what information is not yet known about safety and drug-drug interactions of the novel compound, known risks of receiving the drug(s) and possible alternatives to receiving the drug
Transparency	Should exist at each step for obtaining access, from the application process through drug delivery to reporting of adverse events and outcomes

## MULTI-STAKEHOLDER ACTIONS FOR STRENGTHENING PRE-APPROVAL ACCESS PROGRAMS

Multiple actors need to act urgently on several fronts to develop better pre-approval access programs for TB. Some key steps for different groups are given in Table 2. These pre-approval access programs should be considered an essential supplement to phase III trials that include people with XDR-TB (and other difficult-to-treat forms of TB), as all too often these individuals are excluded from formal controlled studies. There is also a need for robust post-approval access programs to ensure there is bridging between the approval of a drug by a national regulatory authority and its actual routine availability in the national program.

**TABLE 2:** Responsibilities and examples of targeted actions for TB stakeholders for pre-approval access

Stakeholder	Responsibilities and examples of targeted actions
Companies/sponsors	Dedicate funding to and launch pre-approval access programs for products that show evidence of safety and efficacy in phase II studies. These programs should be transparent and easy to navigate
Regulators	Establish or refine regulatory frameworks that enable pre-approval access to novel TB drugs and standards for monitoring and reporting any data collected through these programs. Encourage drug and research sponsors to pursue more inclusive clinical trials and indications
Donors	Provide funding for pre-approval access programs, especially when supporting other aspects of novel TB drug development
National TB Programs and/or Ministries of Health	Ensure there is a framework for and clinician training on pre-approval access as part of national program management of DR-TB, including eligibility criteria, monitoring requirements, packages of holistic support, and outcome reporting
Clinicians and scientists	Support pre-approval access applications with data and other forms of expertise, and share/publish experiences according to national policies
People with DR-TB	Must demand access to novel TB drugs via pre-approval mechanisms, understand the risks and benefits of these products and participate in decision making
All stakeholders	Must learn from the challenges with pre-approval access for other diseases and TB drugs in the past while committing to undertake concerted efforts to avoid repeating these mistakes with future programs. Convene a symposium or international multi-stakeholder meeting to discuss pre-approval access

DR-TB = drug-resistant TB.

## CONCLUSIONS

There are multiple new agents now available for treating multidrug-resistant TB and pre-XDR-TB, but more must be done to improve research and systems of care, especially for people with XDR-TB whose TB is not easy to treat with existing drugs and regimens. If developed in person-centered ways that emphasize the human right to health and dignity for all people with TB, such research and pre-approval access programs can strengthen the overall system of healthcare and have benefits that go beyond saving the lives of individuals with XDR-TB.
